# The Power of Aerostatic Changes upon Abnormal Tissue

**Published:** 1878-12

**Authors:** E. C. Chisholm

**Affiliations:** Tuscaloosa, Ala.


### THE DENTAL REGISTER.

VOL. XXXII.] **DECEMBER, 1878.** NO 12.

### THE POWER OF AEROSTATIC CHANGES UPON ABNORMAL TISSUE.

BY E. S. CHISHOLM, D. D. S., TUSCALOOSA, ALA.

I hope not to be called an egotist in calling attention to a theme comparatively new, and yet as old as the hills; nor eccentric in asking consideration for a short time to a subject at first sight so remote from a professional region. However, new standpoints, "new departures," telephony, phonography, microphony, etc., all conspire to make this a day of advance and research. Besides, an eccentric implies a center as well as diverging extremes. As the fibrous roots of the gigantic oak, diverging in various and remote soils are indispensable to life, health and well being of the tree, so do these extreme departments furnish the necessary pabulum to sustain life, growth and advancement of the great conservative and gigantic trunk of science.

Hence, I will trespass upon your patience in a digression, reflecting, I think, upon so important a theme as diagnosis, which subject can not be too thoroughly amplified upon, nor too frequently impressed; for upon its broad basis is reared the whole structure of the healing art.

But the subject to which I allude directly is, the effect of aerostatic changes upon the human system and especially upon the dental tissues in abnormal conditions.

In an article of mine published some two years since, I barely alluded to this subject, which I hoped might elicit some attention from the profession, but it seems to have passed unheeded. I did the same before the Tennessee State Dental Society, the transactions of which received publication through the DENTAL REGISTER with like results.

This subject, although affording a vast field for investigation, I am only prepared to lay before you the results of restrained efforts, although through a period of six years have I been directly and indirectly observing the potency of the atmosphere upon diseases, and still hope at some future day to be able to offer the subject to you, attested by a series of experiments proving the facts therein contained; but a want of the necessary physical instruments and requisite time, have embarrassed my efforts till the present. Nor with a medium amount of effort have I been able to procure any data of consequence upon this theme, either from dentist or physician, until I am of opinion this subject has been almost entirely ignored, or has failed to receive that attention its importance demands.

The probable cause of this is, that the fields of dental literature are so illimitable and diverse, that it has been rather overlooked. Another, may be, that should we recognize the weather as a diagnostic, no therapeutic agent is at hand. Or in other words, the recognition of the weather as influencing disease—since we can not change the weather, we possess no power to relieve conditions.

The atmosphere and gases it contains have long been known as exerting powerful influence upon the system. The therapeutic power of oxygen has been known over a century Prof. J. L. Cabell, M. D., of the University of Virginia, regards oxygen gas as a valuable agent in all diseases involving defective respiration, and as a tonic to improve assimilation in chronic diseases. We might say much upon the influences of carbon, carburetted hydrogen, nitrogen, and their influences upon the economy in separate states and the various combinations, but admitting their relative potency, it is directly to the atmosphere as a whole, and its changes, that we think the phenomena upon the physique mainly due.

A very short experience either in the practice of medicine or dentistry will be sufficient to convince any thinking person of the potency af the atmosphere in this respect. My earliest observation on this subject was during my connection with the army, where it became my duty to relieve toothache among the soldiers, when frequently on forced marches and without physician's aid. It required no scanning observation to see a greater number of sufferers one day over that of another. Several days would pass without any complaint, when another day would drive dozens for relief.

On these days, too, the hospitals would report a large per cent on the sick list, with neuralgia, cephalalgia, indigestion, diarhœa, rheumatism, asthma, painful corns and bunions, old gun shot wounds and chronic diseases of every order. In my subsequent office practice there has always been an increased demand for my services on certain days. Sometimes comparatively nothing to do—at other times, thronged with callers. Many driven to me merely to have their teeth examined on account of only a shadow of unpleasant sensation—others worse, and so on to swollen jaws and abscessed roots.

These facts prove very conclusively that there exists causes in the atmosphere to which the system is very susceptible. Now, what that is, and how the effect is produced is the question for our solution.

The prominent and noticeable changes which frequently occur in the atmosphere are Thermal, Humid, Electrical and Aerostatic.

These four, while distinct in their respective charactes are so closely allied in many conditions that it would seem at first sight to baffle the most cautious observer to tell from *effects* wherein they play functionary parts in *causes*. That a heated south wind freighted with moisture, at the same time producing barometric full and with a probable increase of electri city may all occur at almost the same moment, there can be no question. In fact, any one of these or all combined may exist at the same time,

But to deal with these separately. We find that *temperature* increased or diminished, seems not to influence abnormality, for we have diseases of the character above mentioned, in cold or warm seasons, and in torrid, temperate and frigid regions all the same. It is beyond controversy that sudden changes, particularly from warm conditions of the body to that of cold, will produce catarrhal diseases, but these are not the conditions referred to at all. We allude to effects principally upon disease—and which come with periodical weather changes and influencing increased abnormality.

As to the adaptability of the skin to any temperature by which it may change thermotic, it might be interesting to heed the experiments by Dr. Winternitz. He says that the giving off of heat from the system may vary more than sixty degrees downward or ninety-two degrees upward. There is then great power in the cutaneous functions to regulate temperature without producing disease, unless too sudden.

As for *humidity*, the regions of Great Britian, and particularly localities along the coast of Ireland, are characterized for moisture, nor are the rainy and moist seasons of the warmer climates never mentioned, especially as exerting morbific effects upon diseased tissue. Hence, we do not deem moisture a just ground for the cause. Dr. Oliver W. Holmes thinks, on the other hand, humid regions aid physical development, and the dryness of our atmosphere produces diseases unlike those of Ireland and England.

With regard to *electricity* being the cause, I can say but little, having no instruments which could give me any satisfactory registration of electrical changes. But my observation convinces me that the periods of changes of atmosphere, peculiarly suited to effect the system most, precedes lightning and thunder by sometimes a few hour's time, and not unfrequently more than one day. So we must not attribute it exclusively to electricity.

It is to the aerostatic condition that we feel safe in asserting, demands our most serious consideration, and the most probable ground of the increased systemic or local disturbance.

By the word aerostation, we literally mean, "The science of weighing air," and directly refers to its rarity or density, which is indicated by the rising and falling of the barometer.

Meteorological observation teaches that at great altitudes, when the atmosphere is far less dense than at ordinary elevations, the intensity of sound is diminished, and respiration impeded. and the minute veins of the body swell and sometimes open. A pistol shot on Mt. Blanc can only be heard a short distance. Guy Lussac and Biot ascended from Paris two thousand, five hundred feet high, when breathing was painful and difficult; and upon the high table-lands of Peru the lips of Dr. Tschudi cracked and burst, while the blood flowed from his eyelids. In consequence of the reduction of atmospheric pressure, water boils at low temperature. Thus at Quito, nine thousand, five hundred and thirty-seven feet above the sea level, ebullition takes place at the temperature of one hundred and ninety-six F. The system feels vascular and full, and blood flows feebly through the veins, accompanied by a sensation of languor and stupidity. Now this condition, dependent upon altitudes, can be brought about at medium elevations by atmospheric phenomina, producing similar results, though not so intense; and will be indicated by falling barometer.

With average pressure of fifteen pounds to the square inch, (as experiment long since has demonstrated), upon our bodies, and the bodies sustained by an equal power of resistance, wisely provided, it follows that a less pressure would momentarily give a decided and sensible change.

A common experiment of the power of this pressure can be made by removing from your vulcanizer, the flask before sufficiently cool, and you will have the satisfaction of seeing such rapid disintegration of the plaster contained, as will allow your plate to get out of shape. This phenomenon will be due to the reduction of the external pressure, while the plaster inside is still under steam heat and pressure.

On the same principle, when we have a nerve exposure and while it is left quiet, and no mechanical violence done, it will be free from pain. As soon, however, as the barome-ric pressure is removed, the nerve is sucked or drawn through the irregular asperities of the edges around the point of ex-posure, and causes absolute change of shape of tissue, and consequently violent harm. All who have ever suffered with an exposed nerve, know the effect of sucking with the tongue. Why not, then, the atmosphere produce similar pressure, or want of pressure?

Should the nerve be devitalized and the nerve canal open, the same result can be obtained. Likewise with an abscess at the end of the root of a tooth. So it is that the foot ex-pands when these attacks of weather induces greater vascu-larity of the system, and the same boot or shoe can not be worn without pain.

Should we eat the same amount of food, we are apt to take on headache, indigestion, followed with diarrhœa.

One might say, even admitting these deductions, that the weather can not be changed, and hence the idea can not be utilized. But to this we would reply that we can use pallia-tives, and reduce inflammatory tendencies. In nerve expos-ure, bridge over them and use materials forming pellicles over the points of exposure. In surgical operations this subject bears no less import.

A report from the Pennsylvania Hospital contains a state-ment of the influence of barometric pressure in surgical ope-rations directed under the care of Dr. Hewson.

He says that fatal results from shock occur in a constant ratio with the dryness of the weather and those suffering from pyemia also bear a direct ratio to the opposite state. On the occasion of two hundred and fifty-nine operations, the barometer was ascending in one hundred and two, de-scending in one hundred and twenty-three, and stationary in thirty-four. Fifty-four of the whole number were fatal; eleven of them were operated on when the barometer was ascending. Thirty-five when descending, and eight when stationary, Of the successful cases, ninety-one were operated on with an ascending barometer, eighty-eight with it descending, and six with it stationary. From these facts, a mortality of not quite 11, (or 10.7), per cent. resulted with an ascending barometer; of over 20, (20.6) per cent. with it stationary; and over 28, (28.4), per cent. with it descending. Hence, by far, the safest time for surgical operations is with an ascending barometer.

These facts, with many others, which we hope subsequently to mention, in a more extended way, attest the importance of attention to the weather, as a diagnostic indication.

The periods of weather we allude to as affecting these changes in the system, occur at all seasons of the year.

After bracing weather, whether cloudy or clear, but more generally the latter, the weather moderates to a little warmer, with probable lowering clouds, and if occurring during the night, will be indicated by fog. Spiders seek this time to spin webs through the fields and hedges. These phenomina too, precede rain or snow, (depending upon season or temperature), sometimes by even twenty-four hours; and invariably produce some of the before mentioned disturbances, when pathological conditions pre-existed.

